# The Impact of PM_2.5_ on the Host Defense of Respiratory System

**DOI:** 10.3389/fcell.2020.00091

**Published:** 2020-03-04

**Authors:** Liyao Yang, Cheng Li, Xiaoxiao Tang

**Affiliations:** State Key Laboratory of Respiratory Disease, Guangzhou Institute of Respiratory Health, The First Affiliated Hospital, Guangzhou Medical University, Guangzhou, China

**Keywords:** PM_2.5_, respiratory system, infection, susceptibility, host defense

## Abstract

The harm of fine particulate matter (PM_2.5_) to public health is the focus of attention around the world. The Global Burden of Disease (GBD) Study 2015 (GBD 2015 Risk Factors Collaborators, 2016) ranked PM_2.5_ as the fifth leading risk factor for death, which caused 4.2 million deaths and 103.1 million disability-adjusted life-years (DALYs) loss, representing 7.6% of total global deaths and 4.2% of global DALYs. Epidemiological studies have confirmed that exposure to PM_2.5_ increases the incidence and mortality of respiratory infections. The host defense dysfunction caused by PM_2.5_ exposure may be the key to the susceptibility of respiratory system infection. Thus, this review aims to assess the impact of PM_2.5_ on the host defense of respiratory system. Firstly, we elaborated the epidemiological evidence that exposure to PM_2.5_ increases the risk of respiratory infections. Secondly, we summarized the experimental evidence that PM_2.5_ exposure increases the susceptibility of different pathogens (including bacteria and viruses) in respiratory system. Furthermore, here we discussed the underlying host defense mechanisms by which PM_2.5_ exposure increases the risk of respiratory infections as well as future perspectives.

## Introduction

**FIGURE 1:**
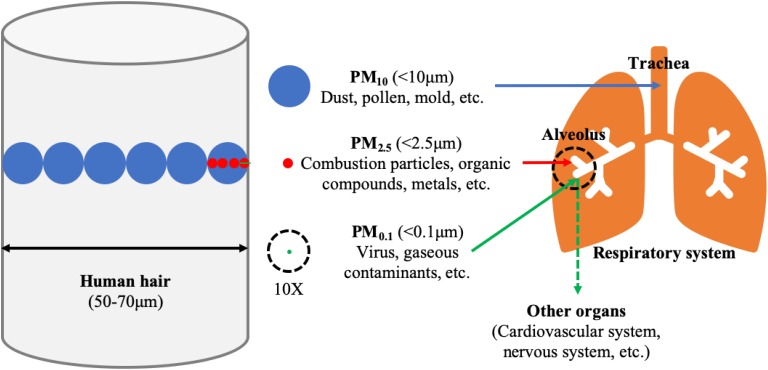
The size, main composition and deposition site in the lung of the particulate matter (PM). The average diameter of human hair is 60 μm, which approximately equivalents to six inhalable particulate matter (PM_10_, <10 μm in diameter, blue color), or twenty-four fine particulate matter (PM_2.5_, <2.5 μm in diameter, red color) or six hundred ultrafine particulate matter (PM_0.1_, <0.1 μm in diameter, green color). Particulate matter, with aerodynamic diameter 2.5–10 μm, is mainly deposited on the trachea. PM less than 2.5 μm in diameter poses the greatest problems, because it can get deep into the terminal bronchioles and alveoli, and some with <0.1 μm in diameter may even get into the bloodstream affecting other organs. 10X: The stuff in the dotted ring is magnified ten times.

Exposure to air pollution, including gaseous pollution and particulate matter (PM) pollution, is a leading contributor to the Global Burden of Disease (GBD 2015 Risk Factors Collaborators, 2016). In recent years, more and more attention has been paid to the impact of PM pollution on public health. PM is a complex mixture of solids and liquids suspended in the air, which can be classified by its aerodynamic diameter as PM_10_ (<10 μm, inhalable particulate matter), PM_2.5_ (<2.5 μm, fine particulate matter) and PM_0.1_ (<0.1 μm, ultrafine particulate matter). PM originates from natural sources (such as dust, sea salt, and wildfires) and anthropogenic emissions (such as vehicles, household wood and coal burning as well as power plants and industry), and the latter accounts for most of the PM pollution (Cho et al., 2018). The components of PM are extremely complex, including inorganic components (such as heavy and transition metals, elemental carbon, and sulfuric/nitric/ammonia salts), organic components (such as polycyclic aromatic hydrocarbons) and biological components (such as fungi, spores, and viruses) (Zhang et al., 2015; Cho et al., 2018). There are certain differences in the source and composition of different types of particulate matter, and the harm to public health varies as well (Figure 1). Among them, PM_2.5_ was considered to be the most harmful one. PM_2.5_ has a large surface area and can adsorb a variety of toxic and harmful substances (Hsu et al., 2016). Because of its small particle size, it can penetrate deep into the lungs and deposit in the terminal bronchioles and alveoli with breath, and even enter the circulatory system through the gas-blood barrier (Pinkerton et al., 2000; Schulze et al., 2017). Exposure to PM_2.5_ can endanger multiple organs in the body, and even lead to systemic adverse effects (Chauhan and Johnston, 2003). Among them, the most common are the respiratory and cardiovascular systems (Xing et al., 2016). This review focuses on the respiratory system, the primary target organ for PM_2.5_ exposure.

PM_2.5_ may be the environmental risk factor that poses the greatest public health hazard. The GBD 2015 ranks PM_2.5_ as the fifth leading risk factor for death, with exposure to PM_2.5_ causing 4.2 million deaths (7.6% of global deaths) and loss of 10.31 million disability-adjusted life-years (DALYs) (4.2% of global DALYs) (Cohen et al., 2017). As claimed by the World Health Organization (WHO), 9 out of every 10 people in urban areas are exposed to high levels of PM_2.5_ (annual average concentration >10 μg/m^3^) from outdoor air pollution, and about 3 billion people using non-renewable fuels are exposed to serious indoor air pollution (World Health Organization [WHO], 2019). According to the Air Quality Life Index (AQLI), sustained exposure to an additional 10 μg/m^3^ of PM_2.5_ reduces life expectancy by 0.98 years^1^. In addition, an increase of 10 μg/m^3^ per day of PM_2.5_ concentration increases 0.29% of overall non-accidental mortality and 0.22% of respiratory disease mortalities (Chen et al., 2017).

A large number of epidemiological studies have shown that PM_2.5_ exposure is closely related to a variety of respiratory diseases (Jo et al., 2017; Wang C. et al., 2019). It is noteworthy that exposure to PM_2.5_ increases the susceptibility to respiratory infections. For instance, numerous clinical studies have found that PM_2.5_ exposure is positively correlated with the number of outpatient visits, emergency visits, and hospitalizations for acute upper or lower respiratory infections (Li et al., 2017; Xia et al., 2017; Strosnider et al., 2019). Related animal models also support the notion that PM_2.5_ exposure increases susceptibility to the lung infection (Yang et al., 2001; Sigaud et al., 2007; Zhao et al., 2014). However, the underlying mechanisms remain elusive. We speculate that PM_2.5_ exposure may impair the host defense of the respiratory system, making the body more susceptible to infection. The purpose of this article is to review the existing epidemiological and experimental evidence to support the above hypothesis and to summarize the possible underlying mechanisms.

## The Epidemiological Evidence

The respiratory system is the primary route for inhaled PM_2.5_. Exposure to PM_2.5_ can cause the development and progression of acute and chronic lung diseases, such as tracheal and pulmonary inflammation (Kampa and Castanas, 2008; Jedrychowski et al., 2013; Ge et al., 2018), asthma and its acute exacerbations (Habre et al., 2014; Zheng et al., 2015), chronic obstructive pulmonary disease (COPD) and its acute exacerbations (Faustini et al., 2013; Tsai et al., 2013). We are concerned with the fact that PM_2.5_ exposure increases the susceptibility to respiratory infections.

Outpatient, emergency, and hospitalization-related data on respiratory infections confirmed that PM_2.5_ exposure was positively associated with the increased respiratory infections. Li et al. (2017) studied the effects of air pollution on outpatients’ acute respiratory outcomes and their study indicated that PM_2.5_ exposure was positively correlated with outpatient visits for upper respiratory tract infection (URTI). A study in the U.S. state of Georgia also showed that pediatric emergency visits for URTI were associated with PM_2.5_ concentrations (Strickland et al., 2016). Another study reported that PM_2.5_ was significantly associated with emergency room visits for respiratory diseases, particularly for URTI and lower respiratory tract infection (LRTI) (Xu et al., 2016). Recently, Strosnider et al. (2019) confirmed that PM_2.5_ exposure was positively correlated with emergency visits for multiple respiratory diseases, including respiratory infections. In addition, Xia et al. (2017) studied the association between different types of air pollution and respiratory hospitalization and found that short-term exposure of PM_2.5_ was positively correlated with the number of hospitalizations for acute respiratory infections. Similarly, another four studies also supported a significant positive correlation between PM_2.5_ and the number of hospitalizations for URTI and LRTI (Belleudi et al., 2010; Li et al., 2018; Liu et al., 2019b; Zhang D. et al., 2019).

The effect of PM_2.5_ exposure on respiratory infections is not immediate, but there is a certain lag effect. For example, PM_2.5_ exposure was positively associated with an increase in hospitalization for acute respiratory infections, but this correlation was delayed by 7–13 days (Xia et al., 2017). Liang et al. (2014) analyzed the time curve of PM_2.5_ concentration and human influenza in Beijing urban area from 2008 to 2011, and proved that there was a significant correlation between PM_2.5_ exposure and influenza, but this correlation showed a 1–2 months delay. In a study of 150,000 cases, Horne et al. (2018) investigated the relationship between PM_2.5_ and the health status of patients with acute LRTI and discovered that the association of PM_2.5_ exposure with respiratory syncytial virus (RSV) infection occurred in 2–4 weeks. The hysteresis effects varied in different studies. Therefore, when investigating the association between PM_2.5_ exposure and respiratory infections, researchers may need to choose the appropriate follow-up time.

Exposure to PM_2.5_ increases the susceptibility to respiratory infections, especially in children and the elderly, as well as the vulnerable groups with hereditary or underlying diseases. A study on the relationship between PM_2.5_ and the health status of patients with acute LRTI revealed that about 77% of the subjects were infants aged 0–2 years. RSV and influenza virus were the main pathogens (Horne et al., 2018). Another study on risks of respiratory infections associated with air pollution in China showed that children under the age of 14 were the predominantly susceptible population of acute respiratory infections caused by air pollution, accounting for 80% of hospitalized cases of respiratory system infections in the study (Xia et al., 2017). Similarly, Liu et al. (2019b) found that students aged 6–17 years were more vulnerable to PM_2.5_ exposure. The maternal exposure to air pollution before birth may result in an impaired lung development and increase the risk of respiratory system infections (Pinkerton and Joad, 2006; Jedrychowski et al., 2013). In addition, by analyzing the association between PM_2.5_ pollution and hospital emergency room visits for total and cause-specific respiratory diseases in urban areas in Beijing, Xu et al. (2016) found that people over 60 years of age demonstrated a higher risk of respiratory disease (including URTI and LRTI) after PM_2.5_ exposure. Exposure to PM_2.5_ is more likely to cause respiratory infections in people with congenital immune deficiencies due to hereditary diseases. For instance, exposure to PM_2.5_ in patients with cystic fibrosis has been reported to be associated with the acquisition of *Pseudomonas aeruginosa* (*P. aeruginosa*). During the follow-up period, each additional PM_2.5_ exposure of 10 μg/m^3^ increased the risk of 24% *P. aeruginosa* acquisition (Psoter et al., 2015). Another study has found that each additional PM_2.5_ exposure of 10 μg/m^3^ increased the risk of methicillin-resistant *Staphylococcus aureus* (MRSA) by 68% (Psoter et al., 2017).

## The Experimental Evidence

*In vivo* studies have shown that as a risk factor for respiratory infection, PM_2.5_ exposure, can prime the lung for greater susceptibility to pathogens by impairing the respiratory host defense. Yang et al. (2001) found that PM exposure suppressed macrophage function and slowed the pulmonary clearance of *Listeria monocytogenes* (*L. monocytogenes*) in rats. Another research discovered that the colony-forming units (CFUs) of *P. aeruginosa* detected in the lung were significantly greater in the PM-exposed mice compared to the control mice (Liu et al., 2019a). Zhao et al. (2014) found that prior PM_2.5_ exposure markedly increased the susceptibility of rats to subsequent *Staphylococcus aureus* (*S. aureus*) infection. Similarly, Duan et al. (2013) found that PM_2.5_ exposure increased the susceptibility of rats to *Klebsiella pneumoniae* (*K. pneumoniae*) infection. Sigaud et al. (2007) and Migliaccio et al. (2013) established an exposure model in mice and subsequently infected with *Streptococcus pneumoniae* (*S. pneumoniae*). They found that PM exposure reduced bacterial clearance in the lungs of mice. In addition, Ma et al. (2017) discovered that exposure to PM_2.5_ lowers influenza virus resistance. We summarized the *in vivo* experimental studies of PM_2.5_ on respiratory host defense (Table 1).

**TABLE 1:** Summary of *in vivo* experimental studies of PM_2.5_ on respiratory host defense.

**Animal species**	**Pathogen**	**Exposure method**	**Effects on respiratory host defense**	**References**
SD rats	Listeria	Intranasal instillation	Exposure to diesel exhaust particles (DEP) decreased the ability of macrophages to produce antimicrobial oxidants in response to Listeria, which may play a role in the increased susceptibility of rats to pulmonary infection	Yang et al., 2001
BALB/c mice	*S. pneumoniae*	Intranasal instillation	The combination of γ-interferon (IFN-γ) priming and concentrated ambient particles (CAPs) exposure led to an inflamed alveolar milieu where oxidant stress caused loss of antibacterial functions in alveolar macrophages (AMs) and recruited polymorphonuclear granulocytes (PMNs)	Sigaud et al., 2007
BALB/c mice	*S. pneumoniae*	Whole-body exposure (smoking)	Exposure to wood smoke-derived particulate matter decreased the ability of pulmonary macrophages to effectively mount a defense against infection, and appeared to be mediated via RelB activation	Migliaccio et al., 2013
SD rats	*K. pneumoniae*	Intranasal instillation	PM_2.5_ exposure increased the susceptibility of the rats to *K. pneumoniae* infection and decreased bacterial clearance. Its mechanism may be related to the impairment of bronchial mucociliary system and interaction of cytokines.	Duan et al., 2013
Wistar rats	*S. aureus*	Intranasal instillation	Exposure to PM_2.5_ increased susceptibility to respiratory *S. aureus* infection in rats via reducing pulmonary natural killer cells and suppressing the phagocytosis ability of AMs.	Zhao et al., 2014
Mice*	Influenza virus	Intranasal inhalation	Long-term exposure to PM_2.5_ lowered influenza virus resistance via down-regulating pulmonary macrophage Kdm6a and mediated histones modification in IL-6 and IFN-β promoter regions	Ma et al., 2017
C57BL/6J mice	*P. aeruginosa*	Intracheal instillation	PM disrupted tight junctions (TJs) via oxidative stress to promote bacterial infection	Liu et al., 2019a

*The specific strain is not indicated in the literature.

*In vitro* experiments have also confirmed that PM_2.5_ exposure increased the susceptibility of respiratory infection. For example, PM_2.5_-pretreated A549 cells have a significantly increased risk of infection with *Mycobacterium tuberculosis* (*M. tuberculosis*) (Rivas-Santiago et al., 2015), and PM can disrupt the airway epithelium through oxidative burst to promote *P. aeruginosa* infection (Liu et al., 2019a). Similarly, Chen et al. (2018) found that PM suppressed airway antibacterial defense, causing an increased susceptibility to *P. aeruginosa*. In addition, adhesion is the key to microbial invasion of the respiratory tract. PM increased the binding of *S. pneumoniae* to both primary alveolar macrophages (AMs) and the murine macrophage cell line J774 A.1 but decreased internalization of bacteria (Zhou and Kobzik, 2007). Mushtaq et al. (2011) have discovered that urban PM increased the adhesion of *S. pneumoniae* to human tracheal epithelial cells. We also summarized the *in vitro* experimental studies of PM_2.5_ on respiratory host defense (Table 2).

**TABLE 2:** Summary of *in vitro* experimental studies of PM_2.5_ on respiratory host defense.

**Cell line**	**Pathogen**	**Culture method**	**Effects on respiratory host defense**	**References**
Murine primary alveolar macrophages and the murine macrophage cell line (J774 A.1)	*S. pneumoniae*	Submerged	Soluble metal, especially iron, in the PM played an important role in the inhibition of macrophage phagocytosis killing of *S. pneumoniae*	Zhou and Kobzik, 2007
A549 cells and Human primary bronchial epithelial cells (HBEpC)	*S. pneumoniae*	Submerged	Urban PM increased adhesion of *S. pneumoniae* to human airway epithelial cells. PM-stimulated adhesion was mediated by oxidative stress and platelet-activating factor receptor (PAFR)	Mushtaq et al., 2011
A549 cells	*M. tuberculosis*	Submerged	Exposure of A549 cells to PM induced cellular senescence, a likely cause of the observed downregulation of HBD-2 and HBD-3 and the subsequent loss of *M. tuberculosis* growth control	Rivas-Santiago et al., 2015
BEAS-2B	*P. aeruginosa*	Submerged	PM impaired airway epithelial defense by impeding the induction of HBD-2 via an oxidative burst, potentially causing an increased susceptibility to infection	Chen et al., 2018
BEAS-2B	*P. aeruginosa*	Submerged	PM disrupted tight junctions (TJs) via oxidative stress to promote bacterial infection	Liu et al., 2019a
				

## Possible Mechanisms

**FIGURE 2:**
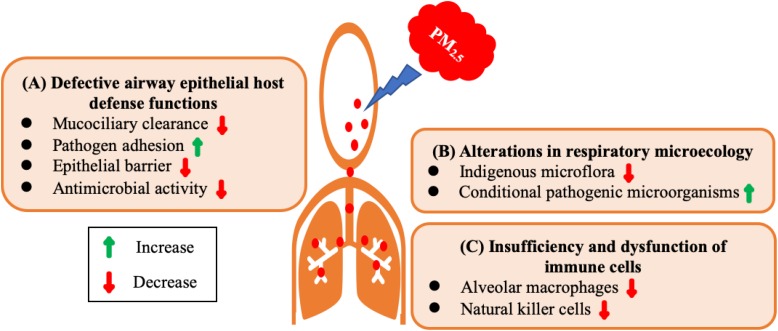
Possible mechanisms underlying defective host defense of respiratory system by PM_2.5_ exposure. **(A)** Defective airway epithelial host defense functions. PM_2.5_ exposure increases adhesion of pathogens to epithelial cells, impairs epithelial barrier function, compromises mucociliary clearance, and reduces antimicrobial activity. **(B)** Alterations in respiratory microecology. PM_2.5_ exposure decreases indigenous microflora and increases the content of conditional pathogenic microorganisms. **(C)** Insufficiency and dysfunction of immune cells. PM_2.5_ exposure decreases the number of natural killer cells and impairs the phagocytic capacity of alveolar macrophages.

PM_2.5_ exposure impairs the host defense of respiratory system causing the body more susceptible to infection. We dissect the underlying mechanisms from the following three aspects: defective airway epithelial host defense functions, alterations in respiratory microecology, insufficiency and dysfunction of immune cells (Figure 2).

### Defective Airway Epithelial Host Defense Functions

**FIGURE 3:**
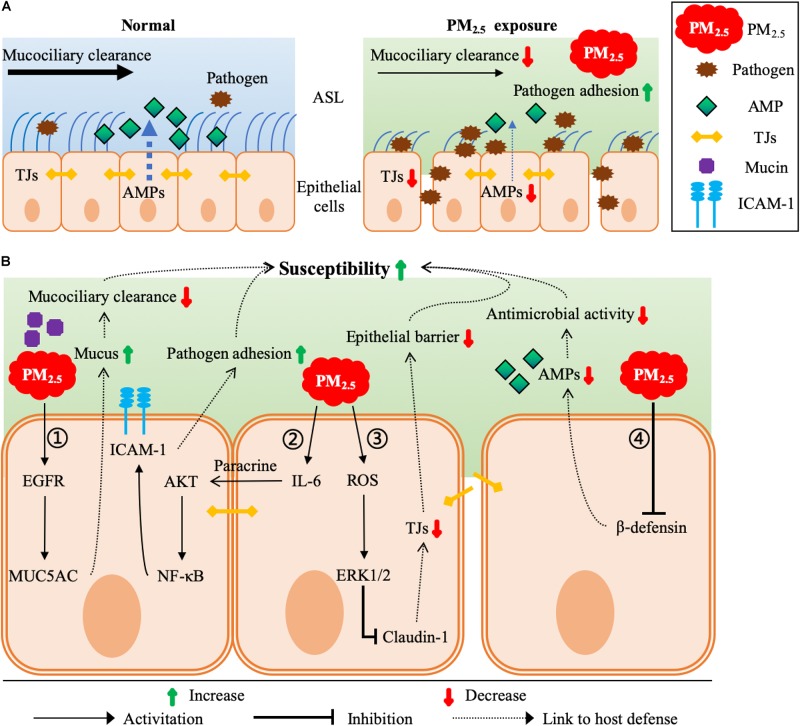
Molecular mechanisms underlying PM_2.5_-induced defective airway epithelial host defense functions. **(A)** Major host defense functions of normal and PM_2.5_-exposured airway epithelia. Normal airway epithelia are protected from pathogens mainly by mucociliary clearance, the barrier functions of the epithelium, and the secretion of a number of AMPs with antimicrobial activities. However, PM_2.5_ exposure disrupts these host defense functions, resulting in more pathogens in the airways. **(B)** Underlying molecular mechanisms: ① **PM_2.5_ → EGFR ↑→ MUC5AC ↑→ Mucus ↑→ Mucociliary clearance ↓**: PM_2.5_ up-regulates the expression of MUC5AC (one of the predominant mucins produced by the airway epithelium) by activating EGFR pathway, resulting in decreased mucociliary clearance. ② **PM_2.5_ → IL-6 ↑→ AKT ↑→ NF-κB ↑→ ICAM-1 ↑→ Pathogen adhesion ↑**: PM_2.5_ activates AKT/NF-κB pathway through IL-6 paracrine signaling, which then up-regulates the expression of ICAM-1 (an important glycoprotein on the cell surface) in the lung to increase the adhesion of pathogens to the airway epithelium. ③ **PM_2.5_ → ROS ↑→ ERK-1/2 ↑→ Claudin-1 ↓→ TJs ↓→ Epithelial barrier ↓**: PM_2.5_ down-regulates the expression of claudin-1 (a major structural protein of TJs) via generating ROS and activating ERK1/2 pathway, resulting in more pathogens to translocate across the disrupted epithelial barrier. ④ **PM_2.5_ →β-defensin ↓→ AMPs ↓→ Antimicrobial activity ↓**: PM_2.5_ inhibits the expression and secretion of β-defensin (one of the major AMPs in ASL), which allows more pathogens to survive and exacerbates respiratory infections.

At the interface between the external environment and the host, the airway epithelium serves as the first line of host defense against pathogens. The airway epithelial host defense functions mainly include mucociliary clearance, the barrier functions of the epithelium, and the secretion of a number of proteins and peptides with antimicrobial activities (Figure 3A). Mucociliary clearance in the airway epithelium is a critical protective function and is essential for the clearance of respiratory pathogens. In general, most foreign bodies inhaled into the lungs can be removed in time by the mucociliary clearance system. However, it has been reported that PM_2.5_ exposure decreased bacterial clearance by impairing the bronchial mucociliary system (Duan et al., 2013). Mucin hyperproduction or hypersecretion is a common reason for decreased mucociliary clearance. Val et al. (2012) found that the expression of MUC5AC, one of the predominant mucins produced by the airway epithelium, was upregulated via the epidermal growth factor receptor (EGFR) pathway after PM_2.5_ exposure in mice (Figure 3B).

The adhesion of pathogens to host cells is a prerequisite for infection. A study has reported that exposure to urban PM increased the adhesion of *S. pneumoniae* to human airway epithelial cells, and the addition of N-acetylcysteine (NAC, an antioxidant) reversed this process, possibly be related to reactive oxygen species (ROS) produced by oxidative stress (Mushtaq et al., 2011). In addition, Liu et al. (2018) reported that ROS induced by PM_2.5_ activated the AKT/STAT3/NF-κB pathway through IL-6 paracrine signaling, which then upregulated the expression of intercellular adhesion molecule-1 (ICAM-1, an important glycoprotein on the cell surface) in the lung to increase the adhesion of pathogens to the airway epithelium (Figure 3B). Woo et al. (2018) also found that PM_2.5_ could enhance the adhesion of *P. aeruginosa* to epithelial cells, the mechanism of which depended on the increased bacterial surface hydrophobicity and damaged human cell plasma membrane by PM_2.5_.

Tight junctions (TJs) are the significant protein complexes at cell-cell interfaces that connect adjacent cells with each other to form lung epithelial barrier against pathogens (Schlingmann et al., 2015). Lack of an intact TJs structure, the airway epithelial barrier cannot keep tight. It will allow pathogens to translocate across the barrier, making the lungs more susceptible to infection. A recent study reported that PM impaired TJs of airway epithelial barrier via oxidative stress to promote *P. aeruginosa* infection (Liu et al., 2019a). Claudin-1 is a major structural protein of TJs. Similarly, another study also discovered that exposure to PM downregulated claudin-1 expression in human airway cells via the ERK1/2 signaling pathway (Kim et al., 2017) (Figure 3B).

The airway epithelial cells are covered with a very thin fluid layer (airway surface liquid, ASL), which is an important component of the respiratory innate immunity. Antimicrobial peptides (AMPs) content is a significant and indispensable factor affecting the antibacterial effect of ASL. AMPs include salivary agglutinin (SAG), beta-defensins, lactoferrin, secretory IgA, and surfactant protein D (SPD) (Fabian et al., 2012; Kendall et al., 2013; Vargas Buonfiglio et al., 2018). Zhang S. et al. (2019) found that PM_2.5_ exposure attenuated the antibacterial activity of airways by down-regulating the expression of SAG. In addition, several studies indicated that PM_2.5_ exposure down-regulated airway β-defensin expression levels through oxidative stress (Rivas-Santiago et al., 2015; Vargas Buonfiglio et al., 2017; Chen et al., 2018). Collectively, these studies suggested that PM_2.5_ could compromise the host defense function of airway epithelial cells by downregulating the expression of AMPs (Figure 3B).

### Alterations in Respiratory Microecology

In healthy humans, the low respiratory tract is usually sterile, and without permanent bacterial colonization, while the upper respiratory tract (especially oropharynx) has a normal bacterial flora, which is also an important component of respiratory tract’s natural immune defense, providing a biological barrier against foreign matter or pathogenic microorganisms througha space-occupying effect, nutritional competition, and secretion of bacteriostatic or bactericidal substances (Akata et al., 2016; Marsh et al., 2016; Wang L. et al., 2019). The oropharyngeal microecosystems of rats changed following PM exposure, including a decline of indigenous microflora and an increase of the content of conditional pathogenic microorganisms (Xiao et al., 2013). By analyzing 16S rRNA sequencing of respiratory tract lavage fluid, a recent study confirmed that there is a link between PM_2.5_ exposure and alterations of the respiratory tract microecology (Yang et al., 2019).

### Insufficiency and Dysfunction of Immune Cells

A variety of immune cells are resident in the respiratory tract, including AMs, polymorphonuclear granulocytes (PMNs), lymphocytes, etc. Their numbers and functions are essential to protect against pathogen invasion. Zhao et al. (2014) found that the reduction of phagocytic phagocytosis caused by PM_2.5_ exposure was related to a decrease of NKs in a PM_2.5_ exposure rat model and subsequently infected with *S. aureus*. Another research found that PM_2.5_ exposure can trigger a Th2-type immune response and reduce the phagocytic capacity of AMs, which may be related to Toll-like receptor 2 (TLR2) and Toll-like receptor 4 (TLR4) (Zhao et al., 2012). In addition, Ma et al. (2017) found that long-term exposure to PM_2.5_ lowered influenza virus resistance via down-regulating pulmonary macrophage Kdm6a and mediated histones modification in IL-6 and IFN-β promoter regions. Another two studies indicated that PM_2.5_ can be used as an immunity inhibitor to reduce the phagocytic capacity of macrophages, thereby increasing the susceptibility to *S. pneumoniae* infection (Migliaccio et al., 2013; Zhao et al., 2014). PM exposure has also been indicated to decrease the ability of macrophages to produce antimicrobial oxidants in response to *L. monocytogenes*, which may play a role in the increased susceptibility of rats to respiratory infection (Yang et al., 2001).

## Conclusion and Future Perspectives

In summary, there are sufficient epidemiological evidences from outpatient, emergency and hospitalization-related data that exposure to PM_2.5_ increases susceptibility to respiratory infections. However, studies on the association between PM_2.5_ exposure and age, gender, and specific pathogens remain controversial. In the future, a meta-analysis of existing research can be attempted to further confirm the susceptible population of PM_2.5_ exposure to respiratory infections. We notice that there is a lag effect in the association between PM_2.5_ exposure and respiratory infections. This may be due to the variations of the average incubation period of different pathogens. We also note that only a few epidemiological studies have reported the relationship between PM_2.5_ exposure and the infection rates of specific pathogens associated with respiratory infections. Is there a specific pathogen preference for respiratory infections caused by PM_2.5_ exposure? This still requires the unremitting efforts of the researchers.

*In vivo* and *in vitro* studies have shown that PM_2.5_ exposure is beneficial to the adhesion, colonization and growth of microorganisms, but it is not conducive to the removal from the body. However, most of the current *in vivo* experiments establish a PM_2.5_ exposure animal model by intratracheal instillation. This model usually affects the lower respiratory tract and is prone to uneven distribution in the lung lobes. Concentration and enrichment of PM_2.5_ aerosol combined with the oral-inhalation or whole-body exposure system may be the best model for simulating human exposure. In recent years, an emerging technology has appeared, combing versatile aerosol concentration enrichment system (VACES) with oral-inhalation or whole-body exposure system. The advantages and disadvantages of these two models are shown in Table 3. Furthermore, current *in vitro* experiments are limited to epithelial cells- and alveolar macrophage-mediated innate immune responses, lacking attention to adaptive immune responses. Either *in vivo* or *in vitro*, the exposure doses currently used in the study are usually based on the corresponding toxicity experiments. How can such exposure levels represent real-world or clinical scenarios?

**TABLE 3:** Strengths and weaknesses of intratracheal instillation and versatile aerosol concentration enrichment system (VACES) on PM_2.5_ exposure to experimental rodents.

**Method**	**Intratracheal instillation**	**Versatile aerosol concentration enrichment system (VACES)**	
		
		**Oral-inhalation exposure system**	**Whole-body exposure system**
Equipment cost	Low	High	High
Operation difficulty	High	Low	Low
Animal activity level	Limited	Limited	Unlimited
Dosage	Instilled dosage (mg/kg of body weight or mg/animal)	Defined by the PM_2.5_ concentration (mg/m^3^)	
Deposition	Uneven distribution in the lung lobes	Even distribution in the lung lobes	
Application	Acute model, only affecting the lower respiratory tract	Acute or chronic model, affecting the whole respiratory tract	
Source of PM_2.5_	PM_2.5_ powder is usually obtained through high volume air sampler collection, ultrasonic elution and vacuum freeze drying. Different elution methods have an impact on the composition of PM_2.5_ obtained	PM_2.5_ is collected directly from the air and concentrated for the required exposure concentration. The composition of PM_2.5_ will be affected by the spatial and temporal distribution of the collection sites	

PM_2.5_ exposure may impair the host defense system of respiratory system, making the organism susceptible to infection. In brief, the possible mechanisms include defective airway epithelial host defense functions, alterations of the respiratory tract microecology, and insufficient number or dysfunction of immune cells (Figure 2). However, there is still a lack of robust research on molecular mechanisms. Further efforts are desperately needed to elucidate the underlying mechanisms at the molecular level.

## Author Contributions

XT conceived and designed the manuscript. LY, CL, and XT wrote the manuscript and critically revised it. CL and XT generated the figures. XT provided guidance and edited the manuscript.

## Conflict of Interest

The authors declare that the research was conducted in the absence of any commercial or financial relationships that could be construed as a potential conflict of interest.

## Footnotes

1 https://aqli.epic.uchicago.edu/pollution-facts/?l=en

## Abbreviations

AMPs: antimicrobial peptides
AMs: alveolar macrophages
AQLI: Air Quality Life Index
ASL: airway surface liquid
CFUs: colony-forming units
COPD: chronic obstructive pulmonary disease
DALYs: disability-adjusted life-years
GBD: global burden of disease
ICAM-1: intercellular adhesion molecule-1
*K. pneumoniae*: *klebsiella pneumoniae*
*L. monocytogenes*: *Listeria monocytogenes*
LRTI: lower respiratory tract infection
*M. tuberculosis*: *Mycobacterium tuberculosis*
MRSA: Methicillin-resistant *Staphylococcus aureus*
NAC: N-acetylcysteine
NKs: natural killer cells
*P. aeruginosa*: *Pseudomonas aeruginosa*
PM: particulate matter
PM_10_: inhalable particulate matter
PM_2.5_: fine particulate matter
PM_0.1_: ultrafine particulate matter
PMNs: polymorphonuclear granulocytes
ROS: reactive oxygen species
RSV: respiratory syncytial virus
*S. aureus*: *Staphylococcus aureus*
*S. pneumoniae*: *Streptococcus pneumoniae*
SAG: salivary agglutinin
SPD: surfactant protein D
TJs: tight junctions
TLR2: Toll-like receptor 2
TLR4: Toll-like receptor 4
URTI: upper respiratory tract infection
VACES: versatile aerosol concentration enrichment system
WHO: World Health Organization.
